# Invasive and noninvasive markers of human skeletal muscle mitochondrial function

**DOI:** 10.14814/phy2.15734

**Published:** 2023-06-20

**Authors:** Rodrigo Mancilla, Diego Pava‐Mejia, Nynke van Polanen, Vera de Wit, Maaike Bergman, Lotte Grevendonk, Johanna Jorgensen, Esther Kornips, Joris Hoeks, Matthijs K. C. Hesselink, Vera B. Schrauwen‐Hinderling

**Affiliations:** ^1^ NUTRIM School of Nutrition and Translational Research in Metabolism Maastricht University Medical Center Maastricht The Netherlands; ^2^ Department of Nutrition and Movement Sciences Maastricht University Medical Center Maastricht The Netherlands; ^3^ Exercise Physiology and Metabolism Laboratory (LABFEM), School of Kinesiology, Faculty of Medicine Finis Terrae University Santiago Chile; ^4^ Department of Radiology and Nuclear Medicine Maastricht University Medical Center Maastricht The Netherlands; ^5^ Institute for Clinical Diabetology, German Diabetes Center Leibniz Institute for Diabetes Research at Heinrich Heine University Düsseldorf Düsseldorf Germany; ^6^ German Center for Diabetes Research (DZD e.V.) München‐Neuherberg Germany

**Keywords:** human skeletal muscle, mitochondrial function, skeletal muscle mitochondrial respiration

## Abstract

Mitochondria are organelles that fuel cellular energy requirements by ATP formation via aerobic metabolism. Given the wide variety of methods to assess skeletal muscle mitochondrial capacity, we tested how well different invasive and noninvasive markers of skeletal muscle mitochondrial capacity reflect mitochondrial respiration in permeabilized muscle fibers. Nineteen young men (mean age: 24 ± 4 years) were recruited, and a muscle biopsy was collected to determine mitochondrial respiration from permeabilized muscle fibers and to quantify markers of mitochondrial capacity, content such as citrate synthase (CS) activity, mitochondrial DNA copy number, TOMM20, VDAC, and protein content for complex I–V of the oxidative phosphorylation (OXPHOS) system. Additionally, all participants underwent noninvasive assessments of mitochondrial capacity: PCr recovery postexercise (by ^31^P‐MRS), maximal aerobic capacity, and gross exercise efficiency by cycling exercise. From the invasive markers, Complex V protein content and CS activity showed the strongest concordance (Rc = 0.50 to 0.72) with ADP‐stimulated coupled mitochondrial respiration, fueled by various substrates. Complex V protein content showed the strongest concordance (Rc = 0.72) with maximally uncoupled mitochondrial respiration. From the noninvasive markers, gross exercise efficiency, VO_2max_, and PCr recovery exhibited concordance values between 0.50 and 0.77 with ADP‐stimulated coupled mitochondrial respiration. Gross exercise efficiency showed the strongest concordance with maximally uncoupled mitochondrial respiration (Rc = 0.67). From the invasive markers, Complex V protein content and CS activity are surrogates that best reflect skeletal muscle mitochondrial respiratory capacity. From the noninvasive markers, exercise efficiency and PCr recovery postexercise most closely reflect skeletal muscle mitochondrial respiratory capacity.

## Graphical abstract

There are numerous methods available to measure human skeletal muscle mitochondrial function and oxidative capacity; however, these various readouts are not always in full agreement with each other. We examined which commonly used invasive (VDAC, mtDNA, TOMM20, CS, and OxPhos) and noninvasive (PCr recovery, VO_2max_, and exercise efficiency) markers of skeletal muscle mitochondrial capacity correlate best with the mitochondrial respiratory capacity in permeabilized human muscle fibers. From the invasive markers, Complex V protein content and CS activity are surrogates that best reflect skeletal muscle mitochondrial respiratory capacity. From the noninvasive markers, exercise efficiency, and PCr recovery postexercise most closely reflect skeletal muscle mitochondrial respiratory capacity. These results are of relevance for studies that aim to investigate skeletal muscle mitochondrial function in humans but do not possess the high‐resolution respirometry methodology and/or are unable to obtain muscle biopsies.

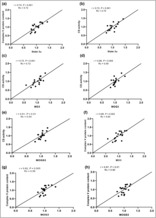

## 1 INTRODUCTION

Mitochondria are organelles that fuel cellular energy requirements by ATP formation via aerobic metabolism and hence determine the oxidative capacity of a cell. Alterations in mitochondrial function will therefore negatively impact energy metabolism and have been associated with a wide variety of metabolic diseases such as obesity‐related insulin resistance (Ritov et al., 2005) and type 2 diabetes (Kelley et al., 2002). Skeletal muscle is the largest and most metabolically active organ in humans with a crucial role in enabling muscle contraction and therefore motion. Due to the limited storage capacity for ATP, human skeletal muscle is highly enriched with mitochondria to sustain contractile activity.

In humans, skeletal muscle mitochondrial function can be determined by a wide variety of invasive measurements, ranging from the assessment of the activity and/or content of (regulatory) proteins and enzymes of mitochondrial metabolism up to the quantification of oxygen consumption and ATP production rates in muscle specimens and/or isolated mitochondria. Furthermore, noninvasive in vivo approaches using magnetic resonance spectroscopy (MRS) have also been applied to determine skeletal muscle oxidative capacity. Thus, in vivo skeletal muscle oxidative capacity can be estimated by measuring the recovery rate of phosphocreatine (PCr) after exercise, using phosphorus magnetic resonance spectroscopy (^31^P‐MRS; Kemp et al., 2015).

While there are numerous methods available to measure skeletal muscle mitochondrial function and oxidative capacity, these various readouts are not always in full agreement with each other. This indicates that other factors may interfere with (some of) the outcome measures and/or that the different markers for mitochondrial function may, partly, reflect different characteristics of mitochondrial metabolism. For instance, it was previously shown that in vivo skeletal muscle oxidative capacity as measured by ^31^P‐MRS was significantly associated with skeletal muscle citrate synthase (CS) activity in young, healthy human volunteers with varying level of physical activity, but not with cytochrome c oxidase (COX); both widely used markers of skeletal muscle mitochondrial function (Larson‐Meyer et al., 2001). Similarly, mitochondrial respiratory capacity from permeabilized muscle fibers was associated with specific markers of skeletal muscle mitochondrial content in young, healthy individuals with a widely different training status (Larsen et al., 2012), such as cardiolipin and CS, but not with mitochondrial DNA copy number (mtDNA; Larsen et al., 2012).

In this study, we aimed to examine which commonly used invasive (VDAC, mtDNA, TOMM20, CS, and OxPhos) and noninvasive (PCr recovery, VO_2max_, and exercise efficiency) markers of skeletal muscle mitochondrial capacity correlate best with the mitochondrial respiratory capacity in permeabilized human muscle fibers. The latter was deemed the most relevant outcome for skeletal muscle oxidative capacity in biopsies, since it encompasses factors such as mitochondrial content, enzymatic activity, and protein content. Therefore, it is interesting to investigate how this parameter is related to other markers of oxidative capacity determined in biopsies and to in vivo measures of oxidative capacity. These associations were examined in young, healthy individuals encompassing a wide range of maximal aerobic capacity based on VO_2max_. Next, to investigate whether there is a linear relationship between variables, we also studied the agreement between parameters by evaluating their concordance.

## 2 MATERIALS AND METHODS

### 2.1 Participants

Participants included in this study were derived from two different studies, both performed at Maastricht University and approved by the Ethics Committee of the Maastricht University Medical Center+. Studies were registered at http://clinicaltrials.gov with identifiers NCT03697928 and NCT03666013. For this study, 19 young, healthy, male volunteers (aged 18–40 years) were included, 13 individuals from the study with identifier NCT03697928, and six individuals from the study with identifier NCT03666013. The study was conducted in accordance with the principles of the Declaration of Helsinki, and all participants provided their written informed consent. Prior to inclusion and after an overnight fast, all participants underwent a medical screening that included a venous blood sample, a resting electrocardiogram (ECG), and a medical history questionnaire. Exclusion criteria were contraindications for MRI examination, uncontrolled hypertension, smoking, and excessive alcohol consumption or drug abuse.

### 2.2 Study design

Participants reported to the university under fasting conditions (5–10 h) on two different days with at least 72 h of rest in between test days. On the first day, subjects performed an incremental cycling test until exhaustion to determine maximal aerobic capacity (VO_2max_; Kuipers et al., 1985). Body composition and total body mass were measured using air displacement plethysmography (BODPOD®, Cosmed; Dempster & Aitkens, 1995). On the second day, a muscle biopsy was taken while resting. Subsequently, phosphorus magnetic resonance spectroscopy (^31^P‐MRS) was performed to measure in vivo skeletal muscle oxidative capacity in *m. vastus lateralis* as previously described (Schrauwen‐Hinderling et al., 2007) and participants performed a submaximal cycling test at 50% of their maximal power output (as assessed during the VO_2max_ test) to determine gross mechanical exercise efficiency. Participants were instructed to maintain their habitual diet and to refrain from any strenuous physical activity during the 3 days directly preceding the test days.

### 2.3 Submaximal cycling test and exercise efficiency

During the submaximal exercise test, oxygen consumption (O_2_) and carbon dioxide (CO_2_) production were measured by indirect calorimetry for at least 10 min. Participants were instructed to maintain a cadence between 60 and 80 rpm throughout the test. To calculate the energy expenditure upon exercise, the Weir equation (Weir, 1949) was used from the measurements of O_2_ consumption and CO_2_ production. Gross energy efficiency (GEE) was calculated as the ratio of power output (watts converted in kJ/min) over exercise energy expenditure (EEE; kJ/min) and expressed as percentage, as previously reported (Matomäki et al., 2019):

$$\mathrm{GEE}\;\left(\%\right)=\left(\text{Work}\;\left(\mathrm{kJ}/\min\right)/\mathrm{EEE}\;\left(\mathrm{kJ}/\min\right)\right)\times 100$$

### 2.4 Skeletal muscle biopsy

A muscle biopsy was obtained from the *m. vastus lateralis* according to the Bergström method (Bergström et al., 1967) using a side‐cutting needle under local anesthesia (1.0% lidocaine without epinephrine). A portion of the muscle biopsy was immediately placed in ice‐cold preservation medium (BIOPS, OROBOROS Instruments) and used for the assessment of mitochondrial respiratory capacity in permeabilized muscle fibers. The remaining portion of the muscle biopsy was immediately frozen in melting isopentane and stored at −80°C until further analysis.

### 2.5 Ex vivo skeletal muscle mitochondrial respiration

Permeabilized muscle fibers were prepared from the muscle tissue collected in the BIOPS preservation medium, as described previously (van de Weijer et al., 2015). Subsequently, the permeabilized muscle fibers (~2.5 mg wet weight) were analyzed for ex vivo mitochondrial respiration assessment using high‐resolution respirometry (Oxygraph, OROBOROS Instruments; Hoeks et al., 2010). To prevent oxygen deprivation during the measurement, the respiration chambers were hyperoxygenated up to ~400 μmol/L O_2_. Next, a multisubstrate protocol was used in which different substrates were added consecutively at saturating concentrations. State 2 respiration was measured upon the addition of malate (4 mmol/L) plus octanoyl‐carnitine (50 μmol/L) and hence defined as respiration in the presence of saturating substrate concentrations in the absence of ADP. Subsequently, an excess of 2 mmol/L of ADP was added to determine coupled (state 3) respiration supported by a fatty acid substrate. Coupled (state 3) respiration was then maximized by the subsequent addition of the complex I‐linked substrate glutamate (10 mmol/L) and the complex II‐linked substrate succinate (10 mmol/L). Finally, the chemical uncoupler carbonylcyanide‐4‐(trifluromethoxy)‐phenylhydrazone (FCCP) was titrated to assess the maximal capacity of the electron transport chain (state 3u respiration). The integrity of the outer mitochondrial membrane was assessed by the addition of cytochrome C (10 μmol/L) upon maximally coupled respiration. In case cytochrome C increases oxygen consumption >10%, the measurement was excluded from statistical analysis. All measurements were taken in quadruplicate, and data are expressed per mg wet weight.

### 2.6 Western blot analysis

Western blot analyses were performed in Bioplex‐lysates of human muscle tissue as previously described (Wefers et al., 2020). Equal amounts of proteins were loaded on gradient Bolt 4%–12% gels (Novex, Thermo Fisher Scientific). Proteins were transferred to nitrocellulose with the Trans‐Blot Turbo transfer system (Bio‐Rad Laboratories). The following antibodies and dilutions were used in this study: a cocktail of mouse monoclonal antibodies directed against human OXPHOS (dilution 1:5.000; ab110411, Abcam), as well as antibodies directed against TOMM20 (dilution 1:10.000; aba186734; Abcam), porin/VDAC (dilution 1:1.000; sc‐390,996; 1:5000, Santa Cruz biotechnology). The specific proteins were detected using secondary antibodies conjugated with IRDye680 or IRDye800 and were quantified with the CLx Odyssey Near Infrared Imager (Li‐COR, Westburg).

### 2.7 Quantification of mitochondrial DNA content and citrate synthase activity

Mitochondrial DNA (mtDNA) copy number was determined using quantitative real‐time PCR, based on the TaqMan probe method, as described previously (Phielix et al., 2008). mtDNA copy number was calculated from the ratio of NADH dehydrogenase subunit 1 (ND1) to lipoprotein lipase (LPL; mtDNA/nuclear DNA), as described previously (Kaaman et al., 2007). For citrate synthase (CS) activity, ~10 mg of the muscle tissue sample was cut with a cryostat (−20°C), dissolved in 150 μL cold SET buffer (containing 250 mmoL/L sucrose, 2 mmoL/L EDTA and 10 mmoL/L Tris–HCl, adjusted pH at 7.4) and homogenized. The supernatant was used for the determination of citrate synthase activity according to Shepherd and Garland (Shepherd & Garland, 1969). CS activity was expressed as units per gram of protein.

### 2.8 Magnetic resonance spectroscopy

All magnetic resonance spectroscopy (MRS) experiments were performed on a 3T MRI scanner (Achieva 3T‐X; Phillips Healtcare). Food intake was standardized by offering participants a light lunch at noon and asked them to refrain from food until completion of the test day. At 17.00 h, participants were positioned in the MRI scanner and ^31^Phosphorus Magnetic Resonance Spectroscopy (^31^P‐MRS) was performed to measure in vivo skeletal muscle oxidative capacity in m. vastus lateralis as previously described (Schrauwen‐Hinderling et al., 2007), using a 6‐cm surface coil. A series of 150 unlocalized ^31^P‐spectra were acquired using the following parameters: single acquisition (NSA = 1); repetition time (TR) = 4000 ms; spectral bandwidth = 3000 Hz; number of points = 1024. Of the series of 150 spectra, 10 spectra were acquired at rest, 70 spectra acquired during one‐legged knee extension and flexion exercise, and 70 spectra during recovery. The exercise was performed inside the scanner using a custom‐built device with an adjustable weight. The exercise intensity was chosen to correspond to 50%–60% of the one‐legged exercise capacity (determined on a separate day). Spectra were analyzed with a custom‐built MATLAB script (MATLAB 2018a, Mathworks Inc). Inorganic phosphate (Pi), PCr and ATP peaks were fitted, and pH was determined. The PCr recovery was fitted with a mono‐exponential function and the rate constant (κ in s^−1^) was determined as previously reported (Schrauwen‐Hinderling et al., 2007). The rate constant κ of PCr resynthesis is almost entirely dependent on ATP produced by oxidative phosphorylation, hence can be used as a parameter of in vivo oxidative capacity (Kemp & Radda, 1994).

### 2.9 Statistical analyses

Participant characteristics are reported as mean ± standard deviation. Data are presented for the 19 individuals unless otherwise indicated in the table/figure legends. Statistical analysis was performed using SPSS, version 21.0 (IBM Corp.). Shapiro–Wilk normality test was carried out to evaluate normal distribution. Two‐sided Pearson's correlation was calculated between the ex vivo mitochondrial respiratory capacity and the invasive and noninvasive markers of mitochondrial oxidative capacity. For the Pearson's correlations that revealed significance, we subsequently examined the concordance using the Lin's concordance test, which takes into account the variation of the individual data from the line of identity. Lin's scale is between 0 and 1, where 0 indicates no concordance and 1 indicates perfect concordance. This analysis was performed using relative data, as individual values were related to the mean value in all measures. A *p* value <0.05 was set to be statistically significant.

## 3 RESULTS

### 3.1 Participant characteristics

The participants' characteristics are shown in Table 1. It is important to note that the participants included in the current study presented a wide range of maximal aerobic capacity, from relatively untrained up to trained individuals (Thompson et al., 2013). The other characteristics further classify the participants as young and generally healthy, although also body weight, BMI, and body composition displayed a rather wide range.

**TABLE 1:** Participant characteristics.

Characteristics (*n* = 19)	Mean ± SD	Range
Age (years)	24.7 ± 4.5	20.0–40.0
Body weight (kg)	72.7 ± 9.7	56.9–91.0
BMI (kg/m^2^)	23.7 ± 2.4	19.3–28.0
FM (kg)	16.0 ± 5.8	6.5–28.0
Fat percentage (%)	22.0 ± 7.5	10.4–40.4
FFM (kg)	56.6 ± 9.3	41.3–72.9
Fasting plasma glucose (mmol/L)	5.0 ± 0.3	4.5–5.5
VO_2max_ (mL/kg/min)	43.0 ± 6.8	31.4–55.7
Wmax (watts)	243 ± 66	148–366

*Note*: Data are presented as mean ± standard deviation.

Abbreviations: BMI, body mass index; FFM, fat‐free mass; FM, fat mass; VO_2max_, maximal aerobic capacity; *W*_max_, maximal power output.

### 3.2 Mitochondrial oxygen consumption in permeabilized skeletal muscle fibers

In this study, we assessed the respiratory capacity in permeabilized muscle fibers and used this as our reference outcome for skeletal muscle mitochondrial capacity. The minimal and maximal values (range) of ADP‐stimulated (state 3) mitochondrial respiration, upon the addition of different substrates, as well as the maximally uncoupled mitochondrial respiration (state 3u) are shown in Table 2.

**TABLE 2:** Associations between invasive markers of mitochondrial content/capacity and ex vivo mitochondrial respiration in permeabilized muscle fibers.

	Mean ± SD	Range	State 3						State 3u	
			MO3		MOG3		MOGS3			
			*r*	*p*	*r*	*p*	*r*	*p*	*r*	*p*
MO3 (pmol·mg^−1^·s^−1^)	38.4 ± 6.8	22–50								
MOG3 (pmol·mg^−1^·s^−1^)	57.2 ± 8.0	44–79								
MOGS3 (pmol·mg^−1^·s^−1^)	92.8 ± 11.8	69–114								
State 3u (pmol·mg^−1^·s^−1^)	116.6 ± 20.4	89–165								
mtDNA copy number (ND1/LPL)	5.7 × 10^3^ ± 1.3 × 10^3^	3.63 × 10^3^–7.97 × 10^3^	0.44	0.08	0.32	0.23	0.32	0.23	**0.52**	**0.03**
CS activity (μmol/min/g)	94.2 ± 20	60–137	**0.73**	**0.001**	**0.66**	**0.004**	**0.61**	**0.010**	**0.72**	**0.001**
TOMM20 protein content (AU)	0.99 ± 0.2	0.60–1.4	0.41	0.08	**0.51**	**0.02**	**0.57**	**0.01**	**0.62**	**0.005**
VDAC protein content (AU)	1.00 ± 0.2	0.65–1.4	0.002	0.98	**0.53**	**0.02**	0.43	0.06	0.34	0.14
Complex I protein content (AU)	0.96 ± 0.6	0.14–2.5	**0.45**	**0.05**	**0.53**	**0.01**	**0.60**	**0.006**	**0.68**	**0.002**
Complex II protein content (AU)	0.93 ± 0.4	0.36–1.9	0.14	0.57	0.41	0.08	**0.53**	**0.02**	0.36	0.13
Complex III protein content (AU)	0.93 ± 0.3	0.66–1.5	**0.50**	**0.02**	0.34	0.14	**0.56**	**0.01**	**0.63**	**0.004**
Complex IV protein content (AU)	0.96 ± 0.3	0.40–1.5	**0.46**	**0.04**	**0.48**	**0.03**	**0.57**	**0.01**	**0.56**	**0.01**
Complex V protein content (AU)	1.04 ± 0.3	0.62–1.3	**0.66**	**0.002**	**0.62**	**0.005**	**0.57**	**0.01**	**0.74**	**<0.001**

*Note*: ADP‐stimulated respiration fueled by malate + octanoyl‐carnitine (MO3), by malate + octanoyl‐carnitine + glutamate (MOG3) and by malate + octanoyl‐carnitine + glutamate + succinate (MOGS3). Data for CS activity and mtDNA are missing from two participants due to the limited size of their muscle biopsy. Bold values represent significant correlations.

Abbreviations: AU, arbitrary units; CS, citrate synthase; *r*, Pearson's correlation coefficient; *p*; significance value; SD, Standard deviation; State 3u, maximal FCCP‐induced uncoupled mitochondrial respiration.

### 3.3 Linear correlations between invasive markers of skeletal muscle mitochondrial capacity and ex vivo mitochondrial respiration in permeabilized skeletal muscle fibers

Besides analyzing mitochondrial respiratory capacity in freshly prepared, permeabilized skeletal muscle fibers, part of the same muscle biopsy was frozen for subsequent determination of several markers of mitochondrial content and/or capacity. Of these markers, citrate synthase (CS) activity, TOMM20 protein content, VDAC protein content, and the protein content of structural components of most complexes of the oxidative phosphorylation system (OXPHOS) were significantly associated with the ADP‐stimulated (state 3) mitochondrial respiration upon the addition of different substrates (Table 2). Correlation coefficients (*r*) of these significant associations varied between 0.45 and 0.73. Mitochondrial DNA copy number (mtDNA) was not significantly associated with any measure of ADP‐stimulated (state 3) mitochondrial respiration upon the addition of different substrates. All markers, except for VDAC and complex II protein content, were significantly associated with the maximal FCCP‐induced uncoupled respiration (state 3u) in permeabilized muscle fibers (Table 2). Correlation coefficients (*r*) of the significant associations between the various markers and maximally uncoupled respiration ranged between 0.52 and 0.74.

### 3.4 Concordance between invasive markers of skeletal muscle mitochondrial capacity and ex vivo mitochondrial respiration in permeabilized skeletal muscle fibers

**FIGURE 1:**
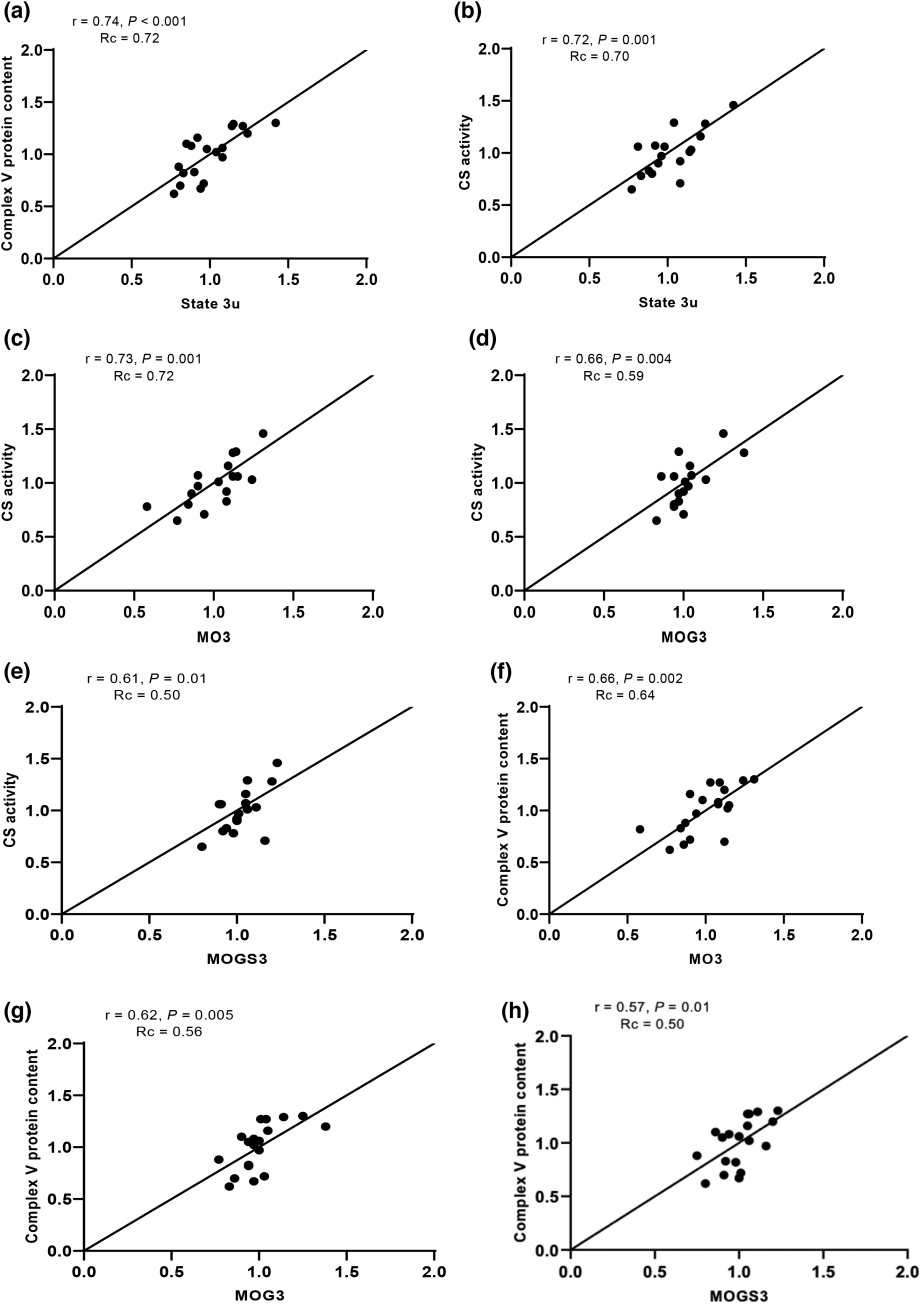
Concordance (or agreement) between mitochondrial respiration in permeabilized muscle fibers and invasive markers of mitochondrial content/capacity (a‐h). Examples with strongest concordance (Rc) are shown. The black lines represent the line of identity (slope = 1, off‐set = 0) between variables.

Next, if a significant linear relationship between parameters was found, we also aimed to test how well the two parameters agreed, in other words, whether the variability in the mitochondrial respiratory capacity in permeabilized muscle fibers is similarly reflected in the various invasive skeletal muscle markers for mitochondrial content/capacity. For this purpose, we computed Lin's concordance coefficient (Rc) for those skeletal muscle markers that were significantly associated with either maximal FCCP‐induced uncoupled mitochondrial respiration (state 3u) or the coupled ADP‐stimulated mitochondrial (state 3). The results of this analysis are displayed in Table 3 and in Figure 1, where the various skeletal muscle markers are listed according to their Rc rank in relation to the maximal uncoupled mitochondrial respiration (state 3u).

**TABLE 3:** Linear correlation coefficients and concordance between invasive markers of mitochondrial content/capacity and ex vivo mitochondrial respiration in permeabilized muscle fibers.

	State 3									State 3u		
	MO3			MOG3			MOGS3					
	*r*	*p*	Rc	*r*	*p*	Rc	*r*	*p*	Rc	*r*	*p*	Rc
Complex V protein content (AU)	0.66	**0.002**	0.64	0.62	**0.005**	0.56	0.57	**0.01**	0.50	0.74	**<0.001**	0.72
CS activity (μmol/min/g)	0.73	**0.001**	0.72	0.66	**0.004**	0.59	0.61	**0.01**	0.50	0.72	**0.001**	0.70
Complex III protein content (AU)	0.50	**0.02**	0.48				0.56	**0.01**	0.47	0.63	**0.004**	0.60
TOMM20 protein content (AU)				0.51	**0.02**	0.46	0.57	**0.01**	0.47	0.62	**0.005**	0.60
mtDNA copy number (ND1/LPL)										0.52	**0.03**	0.49
Complex IV protein content (AU)	0.46	**0.04**	0.38	0.48	**0.03**	0.34	0.57	**0.01**	0.38	0.56	**0.01**	0.43
Complex I protein content (AU)	0.45	**0.05**	0.23	0.53	**0.01**	0.25	0.60	**0.006**	0.25	0.68	**0.002**	0.36
Complex II protein content (AU)							0.53	**0.02**	0.32			
VDAC protein content (AU)				0.53	**0.02**	0.45						

*Note*: ADP‐stimulated respiration fueled by malate + octanoyl‐carnitine (MO3), by malate + octanoyl‐carnitine + glutamate (MOG3) and by malate + octanoyl‐carnitine + glutamate + succinate (MOGS3). Bold values represent significant correlations.

Abbreviations: AU, arbitrary units; CS, citrate synthase; *p*, significance value; *r*, Pearson correlation coefficient; Rc, Lin's concordance coefficient; State 3u, maximal FCCP‐induced uncoupled mitochondrial respiration.

Complex V protein content showed the strongest concordance (Rc = 0.72, Figure 1a) with maximally uncoupled respiration, followed by CS activity (Rc = 0.70, Figure 1b), complex III protein content (Rc = 0.60), TOMM20 protein content (Rc = 0.60), mtDNA content (Rc = 0.49), complex IV protein content (Rc = 0.43), and complex I protein content (Rc = 0.36; Table 3). With respect to maximal ADP‐stimulated respiration, CS activity displayed the strongest concordance (Table 3) upon the different substrate combinations (Rc between 0.50 and 0.72, Figure 1c–e), whereas complex V protein content exhibited the second strongest concordance (Rc between 0.50 and 0.64, Figure 1f–h). TOMM20 protein content, VDAC protein content, and protein content for complex I‐IV of the OXPHOS system exhibited lower concordance values with ADP‐stimulated respiration upon the various substrate combinations, ranging between 0.23 and 0.48 (Table 3).

### 3.5 Linear correlations between noninvasive measures of oxidative capacity and ex vivo mitochondrial respiration in permeabilized skeletal muscle fibers

Because mitochondrial respiratory capacity in permeabilized human muscle fibers is the gold standard, invasive measure of mitochondrial function, we were also interested to investigate how this parameter related to noninvasive readouts for skeletal muscle and whole‐body oxidative capacity. We found that the maximal in vivo skeletal muscle oxidative capacity, expressed by the phosphocreatine (PCr) recovery rate constant after exercise, was significantly associated with MO3 (*r* = 0.81, *p* < 0.001), MOG3 (*r* = 0.62, *p* = 0.008) and the maximally uncoupled respiration (*r* = 0.61, *p* = 0.001; Table 4). Furthermore, also maximal aerobic capacity (i.e., VO_2max_) and gross exercise efficiency were significantly associated with ADP‐stimulated and maximally uncoupled mitochondrial respiration (*r* coefficients between 0.51 and 0.69, all *p* values <0.05; Table 4).

**TABLE 4:** Associations between noninvasive measures of oxidative capacity and mitochondrial respiration in permeabilized muscle fibers.

	Mean ± SD	Range	State 3						State 3u	
			MO3		MOG3		MOGS3			
			*r*	*p*	*r*	*p*	*r*	*p*	*r*	*p*
PCr recovery constant rate [s^−1^]	0.04 ± 0.01	0.025–0.045	**0.81**	**<0.001**	**0.62**	**0.008**	0.43	0.08	**0.61**	**0.001**
VO_2max_ (mL/kg/min)	43.05 ± 7.0	31.4–55.7	**0.54**	**0.01**	**0.51**	**0.02**	**0.61**	**0.005**	**0.54**	**0.01**
Gross exercise efficiency (%)	20.2 ± 2.8	14.6–25.4	**0.54**	**0.01**	**0.62**	**0.005**	**0.62**	**0.004**	**0.69**	**0.001**

*Note*: ADP‐stimulated respiration fueled by malate + octanoyl‐carnitine (MO3), by malate + octanoyl‐carnitine + glutamate (MOG3) and by malate + octanoyl‐carnitine + glutamate + succinate (MOGS3). PCr recovery data is missing in one participant due to implication of the SARS‐CoV‐19 outbreak and one other subject has been excluded from the PCr recovery data analysis due to a pH decline below 6.9. Bold values represent significant correlations.

Abbreviations: AU, arbitrary units; CS, citrate synthase; *p*, significance value; *r*, Pearson correlation coefficient; SD, standard deviation; State 3u, maximal FCCP‐induced uncoupled mitochondrial respiration.

### 3.6 Concordance between noninvasive measures of oxidative capacity and ex vivo mitochondrial respiration in permeabilized skeletal muscle fibers

Similar to the invasive markers for skeletal muscle mitochondrial capacity, we next aimed to determine the agreement of the noninvasive readouts of skeletal muscle and whole‐body oxidative capacity by calculating the Lin's concordance coefficient (Rc) for those measures that significantly associated with ex vivo mitochondrial respiration. The results of this analysis are displayed in Table 5, where the various measures are again ranked according to their Rc value for maximally uncoupled mitochondrial respiration.

**TABLE 5:** Linear correlation coefficients and concordance between noninvasive measures of oxidative capacity and ex vivo mitochondrial respiration in permeabilized muscle fibers.

	State 3									State 3u		
	MO3			MOG3			MOGS3					
	*r*	*p*	Rc	*r*	*p*	Rc	*r*	*p*	Rc	*r*	*p*	Rc
Gross exercise efficiency (%)	0.54	**0.01**	0.53	0.62	**0.005**	0.62	0.62	**0.004**	0.61	0.69	**0.001**	0.67
PCr recovery constant rate [s^−1^]	0.81	**<0.001**	0.77	0.62	**0.008**	0.62				0.61	**0.001**	0.59
VO_2max_ (mL/kg/min)	0.54	**0.01**	0.54	0.51	**0.02**	0.50	0.61	**0.005**	0.60	0.54	**0.01**	0.53

*Note*: ADP‐stimulated respiration fueled by malate + octanoyl‐carnitine (MO3), by malate + octanoyl‐carnitine + glutamate (MOG3) and by malate + octanoyl‐carnitine + glutamate + succinate (MOGS3). Bold values represent significant correlations.

Abbreviations: AU, arbitrary units; CS, citrate synthase; *p*, significance value; *r*, Pearson correlation coefficient; Rc, Lin's concordance coefficient; State 3u, maximal FCCP‐induced uncoupled mitochondrial respiration.

**FIGURE 2:**
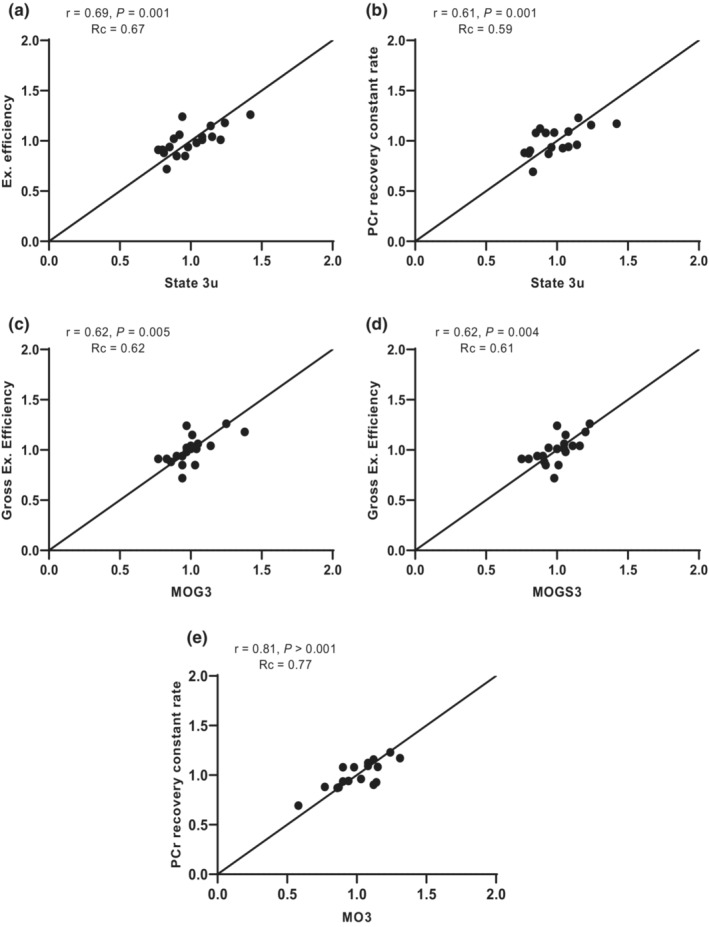
Concordance between mitochondrial respiration in permeabilized muscle fibers and the noninvasive readouts of exercise efficiency (a–d) and PCr recovery rate constant (e) that exhibited the strongest concordance (Rc). The black lines represent the line of identity (slope = 1, off‐set = 0) between variables.

Gross exercise efficiency showed the strongest concordance with maximally uncoupled mitochondrial respiratory capacity (Rc = 0.67, Figure 2a), followed by PCr recovery rate constant (Rc = 0.59, Figure 2b) and maximal aerobic capacity (VO_2max_; Rc = 0.53). For maximally coupled, ADP‐stimulated respiration upon complex I‐ and complex I + II‐linked substrates, gross exercise efficiency also displayed the strongest concordance (Rc = 0.62 and 0.61, respectively, Figure 2c,d, Table 5) followed by PCr recovery rate constant (Rc = 0.62 for MOG3) and VO_2max_ (Rc = 0.50 for MOG3 and Rc = 0.60 for MOGS3, Table 5). PCr recovery rate constant exhibited the strongest concordance with ADP‐stimulated respiration fueled by a lipid substrate (Rc = 0.77, Figure 2e), followed by maximal aerobic capacity (Rc = 0.54) and exercise efficiency (Rc = 0.53).

Finally, we also determined the association and concordance among the noninvasive measures of oxidative capacity (Table S1). PCr recovery postexercise, exercise gross efficiency, and VO_2max_ exhibited significant associations (*r* coefficients between 0.53 and 0.64) with a moderate‐to‐substantial concordance (Rc ranged between 0.51 and 0.63).

## 4 DISCUSSION

We here aimed to examine how well different invasive and noninvasive markers of mitochondrial content/capacity reflect mitochondrial respiratory capacity in permeabilized muscle fibers in young, healthy individuals characterized by a wide range of maximal aerobic capacity. The main findings of the present study were that several markers of skeletal muscle mitochondrial content/capacity determined in muscle biopsy specimens were significantly associated with mitochondrial respiratory capacity in permeabilized skeletal muscle fibers and also showed a fair‐to‐substantial concordance. The invasive markers with the strongest association and strongest concordance with mitochondrial respiratory capacity were protein content for complex V of the OXPHOS system and citrate synthase (CS) activity. In addition, we showed that various noninvasive readouts for skeletal muscle and whole‐body oxidative capacity were significantly associated with mitochondrial respiratory capacity and exhibited a moderate‐to‐substantial concordance. The noninvasive readout with the strongest association with mitochondrial respiratory capacity along with the strongest concordance was gross exercise efficiency, followed by PCr recovery postexercise and maximal aerobic capacity.

### 4.1 Invasive markers of skeletal muscle mitochondrial content/capacity

Classically, the overall volume of the mitochondrial pool is thought to reflect its functional capacity (Glancy et al., 2020). In line with this reasoning, our findings showed that different markers of mitochondrial content mirror skeletal muscle mitochondrial respiratory capacity to a certain extent, with CS activity and protein content of complex V showing the highest level of concordance. Given the relatively small sample size of the present study in combination with the modest difference between these markers, it cannot be concluded from the current study which one is a better reflection of mitochondrial respiratory capacity.

Interestingly, our findings also showed that some invasive markers appear to be more closely related to state 3 respiration, whereas others associate closer with the capacity of the electron transport chain (as reflected by maximally uncoupled respiration). This suggests that different invasive markers of mitochondrial capacity may reflect distinct aspects of mitochondrial metabolism.

Our results are in agreement with a previous study that compared the content and activity of different markers of mitochondrial content and also tested their concordance with the maximal coupled mitochondrial respiration in permeabilized muscle (Larsen et al., 2012). However, that study also indicated that assessment of the enzymatic activity for complex II and complex IV of the OXPHOS system was superior to CS activity, mtDNA copy number, and the enzymatic activity and protein content for complex I, III, and V of the OXPHOS system to reflect maximal coupled mitochondrial respiration in healthy young individuals (Larsen et al., 2012). We also showed that TOMM20, VDAC, and mtDNA are relatively poor proxies in estimating mitochondrial respiratory capacity. In light of the fact that these markers are widely used in the field to explore skeletal muscle mitochondrial adaptations upon exercise training (Balan et al., 2019; Gram et al., 2014; Menshikova et al., 2006), their poor reflection of mitochondrial respiratory capacity is an important and somewhat unanticipated finding.

The poor concordance of TOMM20, VDAC, and mtDNA with mitochondrial respiratory capacity may in part be explained functionally, as these markers of mitochondrial content are not directly involved in the electron transport chain and/or phosphorylation system.

For studies that aim to investigate skeletal muscle mitochondrial respiratory capacity, but do not have the opportunity to measure mitochondrial oxygen consumption or ATP production rates via high‐resolution respirometry methodology, it is essential to make a good choice in regard to the stronger (CS activity and protein content for complex V of the OXPHOS system) and weaker (mtDNA and VDAC protein content) markers of mitochondrial oxidative capacity.

### 4.2 Noninvasive measures of oxidative capacity and ex vivo mitochondrial respiration in permeabilized muscle fibers

We found that noninvasive readouts for skeletal muscle and whole‐body oxidative capacity, such as PCr recovery postexercise, maximal whole‐body aerobic capacity, and exercise efficiency, were significantly associated and exhibited a moderate‐to‐substantial concordance with skeletal muscle mitochondrial respiration. Consistent with our results, prior investigations have documented significant associations between PCr recovery postexercise (Lanza et al., 2011; Layec et al., 2016), maximal whole‐body aerobic capacity (Jacobs & Lundby, 2013), and exercise efficiency (Distefano et al., 2018; Hunter et al., 2019) with mitochondrial respiration from permeabilized muscle fibers. Thus, it was previously shown that the maximal skeletal muscle oxidative capacity, as estimated by PCr recovery postexercise via ^31^P‐MRS, exhibits a strong linear relationship with skeletal muscle mitochondrial respiration and displays a robust test–retest reliability in a similar cohort of human volunteer as in the present study (Lanza et al., 2011). Furthermore, PCr recovery rate constant rate was shown to be significantly associated with citrate synthase activity (McCully et al., 1993).

In addition, improvements in exercise efficiency (Hunter et al., 2019) and maximal aerobic capacity (Pesta et al., 2011) have been shown to parallel increases in skeletal muscle mitochondrial respiration after regular physical activity and exercise training. However, the agreement between exercise efficiency and maximal aerobic capacity with mitochondrial respiration has not been reported before.

Our data extend such previous findings and surprisingly indicate that especially exercise efficiency shows a high degree of agreement with the skeletal muscle mitochondrial respiratory capacity. Furthermore, skeletal muscle oxidative capacity as determined by PCr recovery postexercise was in good agreement and therefore support the widespread use of PCr recovery postexercise as a valid muscle‐specific marker of mitochondrial respiration.

These findings suggest that similar limitations exist in mitochondrial respiration in permeabilized muscle fibers and in vivo function in the current, generally healthy population. In addition, these findings may benefit studies that aim to investigate mitochondrial function but are without the opportunity to collect muscle biopsies.

In the present study, we included young healthy volunteers characterized by a physiological range of maximal aerobic capacity that can be targeted by exercise training interventions. Yet, there was not a perfect concordance between the various invasive and noninvasive markers of mitochondrial capacity with mitochondrial respiration in permeabilized muscle fibers. This indicates that the positive effects of exercise training on skeletal muscle mitochondrial function are not guaranteed to be detected, depending on the selected marker to be analyzed. Certainly, the selection of a poor marker of mitochondrial function can hamper conclusions to be drawn.

Obviously, it should be acknowledged that various differences between the ex vivo and in vivo assessments contribute to the fact that there is no perfect agreement between these markers, that is, these markers may (at least in part) reflect different aspects of mitochondrial metabolism. Thus, when assessing the respiratory capacity of permeabilized muscle fibers, the oxygraph chamber is hyperoxygenated and supplied with excess concentrations of substrates; hence, oxygen and substrate (transport) are not limiting. Moreover, the permeabilization procedure of the sarcolemma further expedites the diffusion of oxygen and substrates into the mitochondria. In vivo, blood flow to the active limbs affects oxygen availability within the contracting muscle tissue, hence affecting the ATP synthesis capacity (Haseler et al., 2007). Conversely, the concentration of substrates and reducing equivalents as well as ADP during exercise in vivo are dictated by the energy stress imposed by contraction and often regulated by intracellular substrate transport. Even the level of concordance among the various noninvasive measures of oxidative capacity was surprisingly modest. Because these noninvasive measures share multiple factors that integrally determine oxidative capacity (e.g., muscle blood flow, mitochondrial content and mitochondrial enzyme activity, oxygen, and substrate transport), we had anticipated a higher level of concordance among these outcomes. These data indicate that factors that underly muscle‐specific oxidative capacity still differ to those that govern whole‐body oxidative capacity. Similarly, these data suggest that factors that underly maximal aerobic capacity still differ to those that determine exercise efficiency.

Several limitations can be identified in the current study. First of all, we only included male volunteers. Future studies are needed to investigate whether similar (or any other) in vivo and in vitro markers of mitochondrial function reflect skeletal muscle mitochondrial respiratory capacity assessed in permeabilizated muscle fibers in women. Also, potential differences in mitochondrial function due to race or disease were beyond the scope of this study; hence, extrapolation of our data to other subject groups needs to be done with care. Furthermore, since we feel that both the in vivo and ex vivo mitochondrial function is dependent on the mitochondrial density and the capacity of individual mitochondria, we did not correct high‐resolution respirometry measurements for mitochondrial content. However, this may mask mitochondria‐specific changes in functional capacity. Please note also that mitochondrial function was defined as the maximal oxygen consumption of mitochondria, while other aspects of mitochondrial bioenergetics, such as efficiency of ATP production were not addressed. A final limitation of the present investigation is the sample size. A difficulty is that a study would require sample sizes of ~200–300 individuals in order to statistically answer the current research question, which makes it unrealistic to be performed. Nevertheless, the present data contribute to the remaining and collective literature aiming to find the best surrogates of skeletal muscle mitochondrial respiratory capacity.

In conclusion, the present study shows that protein content for complex V of the OXPHOS system and CS activity are invasive markers of mitochondrial function that best reflect skeletal muscle mitochondrial respiratory capacity as assessed in permeabilized muscle fibers. Other invasive markers of skeletal muscle mitochondrial density such as mtDNA, TOMM20, VDAC, and protein content for complex I‐IV of the OXPHOS system, represent less accurate surrogates for testing mitochondrial respiration from permeabilized muscle fibers. Moreover, the present study shows that PCr recovery postexercise, maximal aerobic capacity, and exercise efficiency display high agreement with mitochondrial respiration from permeabilized muscle fibers. Exercise efficiency was the noninvasive marker that reflects mitochondrial respiratory capacity best, followed by PCr recovery postexercise and maximal aerobic capacity (VO_2max_). These results are of relevance for studies that aim to investigate skeletal muscle mitochondrial function but do not possess the high‐resolution respirometry methodology and/or are unable to obtain muscle biopsies.

## AUTHOR CONTRIBUTIONS

Rodrigo Mancilla, Joris Hoeks, and Matthijs K. C. Hesselink conceived and designed the study. Rodrigo Mancilla, Joris Hoeks, and Matthijs K. C. Hesselink collected, analyzed, and interpreted the data. Rodrigo Mancilla, Joris Hoeks, and Matthijs K. C. Hesselink wrote the manuscript. Rodrigo Mancilla, Vera B. Schrauwen‐Hinderling, and Matthijs K. C. Hesselink revised and approved the final version of the manuscript. All authors approved the final version of the manuscript.

## FUNDING INFORMATION

The work of R.M is supported by the National Commission of Scientific and Technological Research (CONICYT, Chile) PhD scholarship (Resolucion Exenta number 4426, 2016).

## CONFLICT OF INTEREST STATEMENT

The authors declare that they have not conflict of interest.

## ETHICS APPROVAL

The studies were performed at Maastricht University and approved by the Ethics Committee of the Maastricht University Medical Center+. Studies were registered at http://clinicaltrials.gov with identifiers NCT03697928 and NCT03666013. Participants were given comprehensive, written and verbal information about the experiments, before providing written consent to participate in this study.

## Supporting information


Data S1

## Data Availability

The data that support the findings of this study are available from the corresponding author upon reasonable request.
