# Supplementary material for: Invasive and noninvasive markers of human skeletal muscle mitochondrial function
Source: Physiol Rep. 2023 Jun 20;11(12):e15734. doi: 10.14814/phy2.15734 (PMC10281956; doi:10.14814/phy2.15734)

**Supplementary table 1.** **Linear correlation coefficients and concordance between non-invasive measures of oxidative capacity**

|  | PCr recovery  constant rate [s] | | | Gross exercise efficiency (%) | | | VO2max  (ml/kg/min) | | |
| --- | --- | --- | --- | --- | --- | --- | --- | --- | --- |
|  | r | *P* | Rc | r | *P* | Rc | r | *P* | Rc |
| PCr recovery constant rate [s] |  |  |  | 0.55 | 0.02 | 0.54 | 0.53 | 0.03 | 0.51 |
| Gross exercise  efficiency (%) | 0.55 | 0.02 | 0.54 |  |  |  | 0.64 | 0.003 | 0.63 |
| VO2max  (ml/kg/min) | 0.53 | 0.03 | 0.51 | 0.64 | 0.003 | 0.63 |  |  |  |

r; Pearson correlation coefficient; *P*; significance value; Rc: Lin’s concordance coefficient; CS: citrate synthase; [s]; seconds

**Supplementary table 2.** **Linear correlation coefficients between anthropometric and metabolic variables and invasive and non-invasive measures of oxidative capacity**

|  | State 3 | | | | | | State 3u | | PCr rec.  [s] | | Gross.Ex.  Efficiency (%) | | VO2max  (ml/kg/min) | |
| --- | --- | --- | --- | --- | --- | --- | --- | --- | --- | --- | --- | --- | --- | --- |
|  | MO3 | | MOG3 | | MOGS3 | |  |  |  |  |  |  |  |  |
|  | r | P | r | P | r | P | r | P | r | P | r | P | r | P |
| Body weight (kg) | -0.22 | 0.34 | 0.32 | 0.17 | 0.31 | 0.18 | 0.16 | 0.51 | 0.01 | 0.96 | 0.05 | 0.81 | -0.04 | 0.84 |
| BMI (kg/m^2^) | -0.22 | 0.36 | 0.17 | 0.48 | 0.20 | 0.40 | 0.17 | 0.48 | -0.01 | 0.94 | 0.09 | 0.66 | -0.16 | 0.51 |
| FM (kg) | -0.31 | 0.19 | -0.23 | 0.32 | -0.26 | 0.27 | -0.11 | 0.65 | -0.18 | 0.48 | -0.41 | 0.08 | -0.59 | 0.007 |
| FM (%) | -0.23 | 0.33 | -0.38 | 0.10 | -0.42 | 0.07 | -0.18 | 0.43 | -0.14 | 0.54 | -0.43 | 0.06 | -0.61 | 0.005 |
| FFM (kg) | -0.06 | 0.78 | 0.49 | 0.03 | 0.50 | 0.02 | 0.23 | 0.33 | 0.09 | 0.69 | 0.30 | 0.20 | 0.32 | 0.17 |
| Fasting  Glucose (mmol/l) | -0.42 | 0.07 | 0.11 | 0.63 | 0.04 | 0.85 | -0.18 | 0.44 | -0.18 | 0.49 | 0.04 | 0.86 | -0.09 | 0.71 |

r; Pearson correlation coefficient; *P*; significance value; [s]; seconds

**Supplementary figure 1.** ***Ex vivo* skeletal muscle mitochondrial respiration**


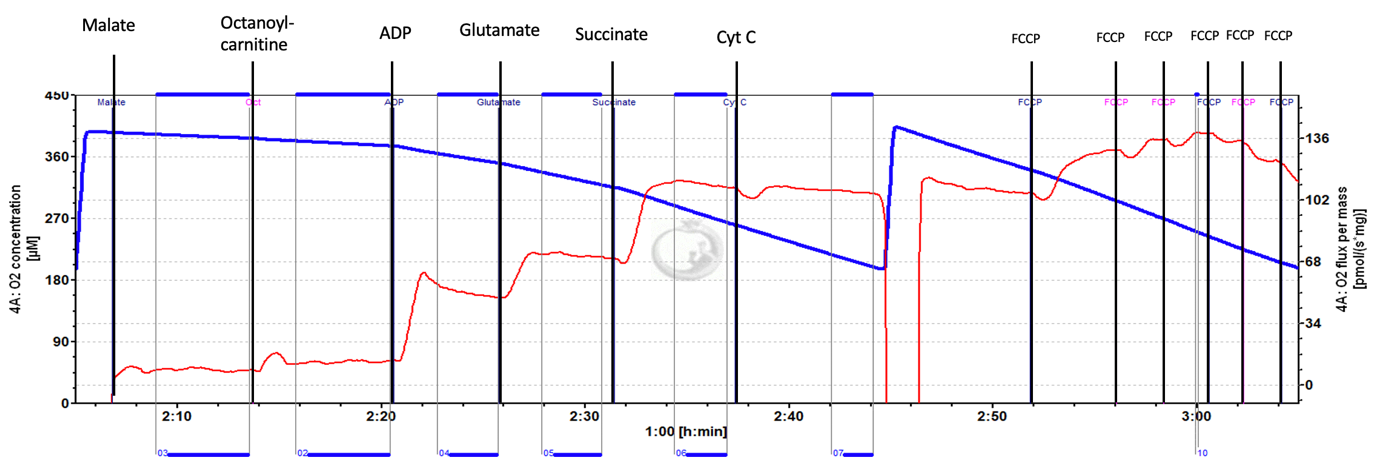


ADP; Adenosyn diphosphate; Cyt C; Cytochrome C; FCCP; chemical uncoupler carbonylcyanide-4-(trifluromethoxy)-phenylhydrazone.

**Supplementary figures 2. Representative pictures of protein quantification by western blot**

1. Quantification of OXPHOS complexes by western blot. The various complexes are depicted in the figure.


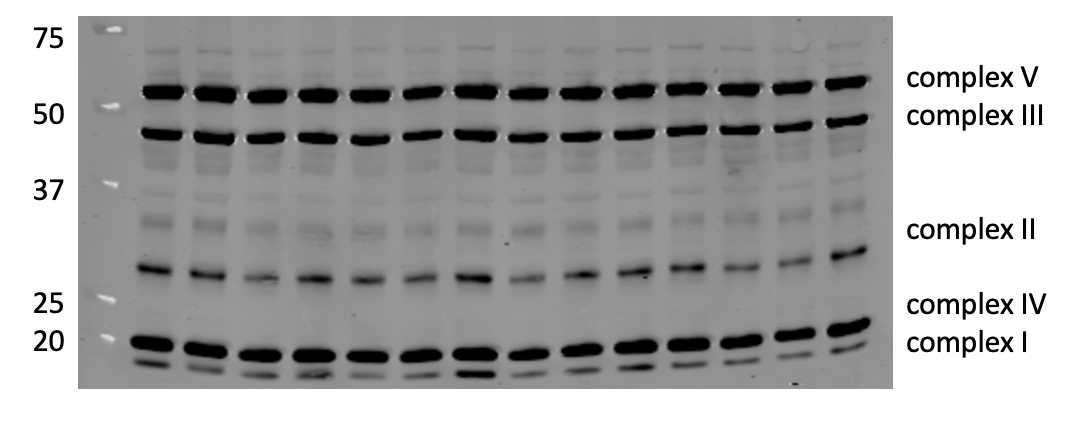


B) Quantification of TOMM20 protein by western blot. The relevant band is labeld.


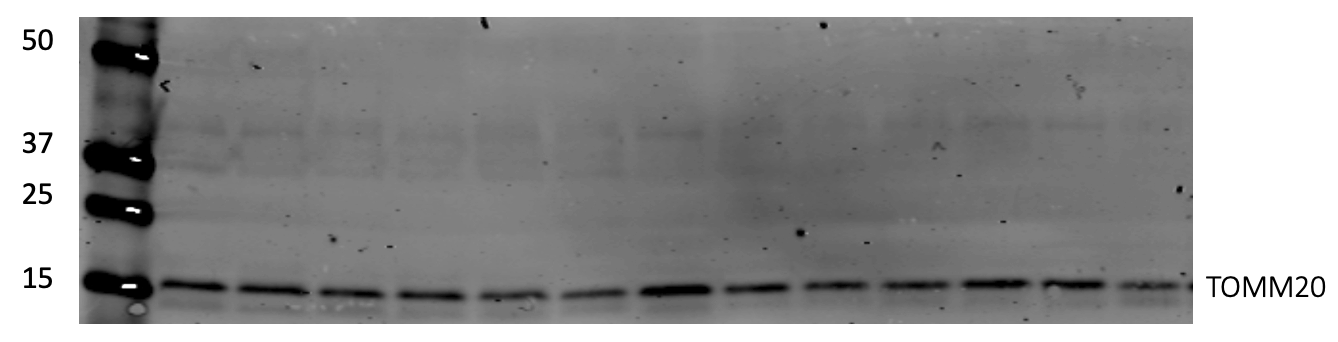


C) Representative picture of quantification of VDAC protein. The relevant band is labeld


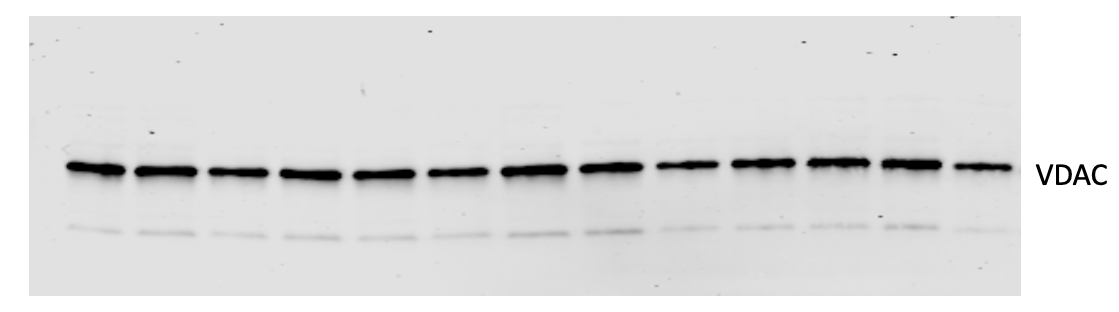

Supplement: Supplementary file 1 — Data S1 [file PHY2-11-e15734-s001.docx]
